# Exploring the mechanism by which modified huangqi chifeng decoction protects podocytes via Arid5a-mediated transcriptional regulation of the AIM2 pyroptosis signaling axis

**DOI:** 10.3389/fimmu.2026.1822567

**Published:** 2026-05-01

**Authors:** Mingming Zhao, Hangyu Duan, Jianing Zhang, Yue Shi, Linghui He, Jianlong Xu, Yundong Yin, Yu Zhang

**Affiliations:** 1 Xiyuan Hospital, China Academy of Chinese Medical Sciences, Beijing, China; 2 Integrated Traditional Chinese and Western Medicine Diagnosis and Treatment Center, Chongqing General Hospital, Chongqing University, Chongqing, China; 3 China Science and Technology Development Center for Chinese Medicine, Beijing, China

**Keywords:** AIM2, Arid5a, modified Huangqi Chifeng decoction (MHCD), podocyte, proteinuria, pyroptosis

## Abstract

**Background:**

Chronic kidney disease (CKD) is a major global health burden with substantial morbidity and mortality. Proteinuria is strongly associated with adverse renal outcomes, and podocyte pyroptosis is increasingly recognized as a key driver of glomerular filtration barrier disruption.

**Objective:**

This study sought to elucidate how Modified Huangqi Chifeng Decoction (MHCD) mitigates renal and podocyte injury by modulating the Arid5a/AIM2-dependent pyroptosis pathway.

**Materials and methods:**

An adriamycin-induced nephropathy (ADR) rat model and an *in vitro* podocyte injury model were established. Twenty-four-hour urinary protein levels, serum biochemical indices, and renal histopathology were assessed. Expression of Arid5a, AIM2, and downstream pyroptosis-related molecules (Caspase-1, ASC oligomerization, GSDMD, IL-1β, and IL-18) was quantified. Mutagenesis of Arid5a binding sites in the AIM2 promoter, along with electrophoretic mobility shift assays (EMSA) and dual-luciferase reporter assays, were used to validate Arid5a’s promoter binding and its transcriptional regulation of AIM2.

**Results:**

MHCD markedly reduced proteinuria, alleviated renal histopathological damage, and improved podocyte viability in ADR rats. These protective effects coincided with downregulation of Arid5a, AIM2, and downstream pyroptosis mediators—including Caspase-1, ASC oligomers, GSDMD, IL-1β, and IL-18. Mechanistically, Arid5a functioned as a transcription factor that binds to the AIM2 promoter to regulate AIM2 expression.

**Conclusions:**

MHCD mitigates renal and podocyte injury by suppressing inflammasome activation and pyroptosis through Arid5a-mediated transcriptional activation of the AIM2 signaling axis.

## 1 Introduction

Chronic kidney disease (CKD) is defined as abnormalities in kidney structure and/or function that persist for at least 3 months and are primarily characterized by a reduction in glomerular filtration rate (GFR) and/or the presence of proteinuria, hematuria, or abnormal renal imaging findings (1). Epidemiological data show that, compared with 1990, the global prevalence of CKD had risen by 29% and CKD-related mortality by 41.5% by 2017 (2). Proteinuria is strongly associated with adverse CKD outcomes; escalating proteinuria levels markedly increase the risk of cardiovascular and renal events as well as all-cause and cardiovascular mortality (3). Podocytes, highly specialized terminally differentiated epithelial cells located on the outer aspect of the glomerular basement membrane (GBM), form the glomerular filtration barrier together with glomerular endothelial cells and the GBM. This barrier permits passage of cationic molecules, electrolytes, and small-to-medium solutes while restricting filtration of anionic and large macromolecules (4). Podocyte injury or loss exposes the underlying GBM, allowing plasma proteins to enter Bowman’s space and subsequently the proximal tubule, thereby generating proteinuria. When podocyte depletion exceeds ~40%, segmental-to-global glomerulosclerosis develops, accompanied by persistent heavy proteinuria and progressive renal functional decline (5).

Research on pyroptosis dates back to 1986. Because early observations revealed caspase dependence, DNA damage, and nuclear condensation—features reminiscent of apoptosis—it was initially misclassified as an apoptotic pathway. Accumulating evidence has since established pyroptosis as a distinct form of pro-inflammatory programmed cell death (6). A major advance in inflammasome biology was the discovery that absent in melanoma 2 (AIM2), a member of the HIN-200 family, functions as a cytosolic double-stranded DNA (dsDNA) sensor that induces caspase-1–dependent maturation of IL-1β. The AIM2 inflammasome comprises AIM2, apoptosis-associated speck-like protein containing a CARD (ASC), and caspase-1, and its activation triggers pyroptotic cell death (7, 8). Although expressed in the kidney, AIM2 acts as a disease-promoting factor; its deletion attenuates unilateral ureteral obstruction (UUO)-induced renal injury, fibrosis, and inflammation by suppressing the inflammasome cytokines IL-1β and IL-18 (9).

Huangqi Chifeng Decoction, originally described in Wang Qingren’s Correction of Errors in Medical Classics (Yilin Gaicuo, Qing Dynasty), has been adapted by Professor Zhang Yu (Xiyuan Hospital, China Academy of Chinese Medical Sciences) into the Modified Huangqi Chifeng Decoction (MHCD). MHCD has been used clinically for more than three decades to manage proteinuria. Preliminary clinical studies indicate that MHCD effectively reduces proteinuria in CKD patients with a favorable safety profile, potentially by mitigating podocyte injury through modulation of podocyte-associated protein expression (10, 11). To elucidate its pharmacological basis, we first conducted a comprehensive chemical analysis of the decoction and drug-containing serum (12, 13). Subsequent studies in an IgA nephropathy rat model demonstrated that MHCD alleviates renal pathological injury and protects podocytes by suppressing TLR4 and IL-17 signaling and reducing downstream pro-inflammatory cytokine expression (14–16). In a complementary adriamycin-induced nephropathy model, MHCD reduced proteinuria and attenuated podocyte damage, characterized by upregulation of the podocyte structural proteins nephrin and podocin, an effect mediated through the inhibition of excessive autophagy (12). Recent evidence implicates the transcription factor adenine-thymine-rich interaction domain 5A (Arid5a) in renal inflammatory injury (17, 18). Combined with our bioinformatic identification of putative AIM2–Arid5a binding sites, these findings prompted investigation of a potential regulatory interaction. To delineate the mechanisms by which MHCD protects podocytes, we examined the role of Arid5a in regulating transcription of the AIM2 inflammasome pathway using *in vivo* and *in vitro* models of adriamycin-induced injury.

## 2 Materials and methods

### 2.1 Drugs and reagents

MHCD consisted of the following crude herbal components: Radix Astragali (30 g), Radix Paeoniae Rubra (10 g), Radix Saposhnikoviae (10 g), Rhizoma Dioscoreae Nipponicae (20 g), Semen Euryales (20 g), Fructus Rosae Laevigatae (10 g), and Herba Hedyotis Diffusae (20 g). All herbs were supplied by the Pharmacy Department of Xiyuan Hospital, China Academy of Chinese Medical Sciences, and processed into a concentrated extract by the hospital’s Preparation Unit.

Telmisartan tablets (Boehringer Ingelheim Ellas A.E.; NMPA approval number J20180016) were purchased from Boehringer Ingelheim International GmbH (Ingelheim am Rhein, Germany). Adriamycin for model induction was obtained from Shenzhen Main Luck Pharmaceuticals Inc. (NMPA approval number H44024359) and Sigma-Aldrich (Lot #L6529). Primary antibodies used in the study included fibronectin (FN, Abcam, #ab268020), laminin (LN, Abcam, #ab11575), collagen IV (Col IV, Abcam, #ab6586), Arid5a (ABclonal, #A25128), AIM2 (UpingBio, #YP-Ab-03685), caspase-1 (Wanleibio, #WL03450), ASC (Wanleibio, #WL02462), GSDMD (Affinity, #AF4012), IL-1β (Wanleibio, #WL00891), and IL-18 (Wanleibio, #WL01127).

Drug-containing serum was prepared using established pharmacological procedures. Male Sprague-Dawley rats (180–220 g) were acclimated for 1 week under standard laboratory conditions and then randomly assigned to four groups: a low-dose MHCD-containing serum group, a medium-dose MHCD-containing serum group, a high-dose MHCD-containing serum group, and a vehicle control group. All herbs were decocted and concentrated by the Preparation Unit of Xiyuan Hospital. The final preparations were standardized according to the crude-herb equivalent of the whole prescription and prepared at concentrations of 1.25, 2.5, and 5 g crude herbs/mL for the low-, medium-, and high-dose groups, respectively. MHCD was administered by oral gavage once daily for 7 consecutive days at doses of 12.5, 25, and 50 g/kg/day, respectively, calculated on the basis of the crude-herb equivalent of the whole prescription. Thus, the dose of MHCD used in this study was calculated based on the total crude-herb equivalent of the combined formula rather than a single purified compound. Rats in the control group received an equivalent volume of purified water.

### 2.2 *In vivo* experiment in adriamycin-induced nephropathy rats

Fifty healthy male Sprague–Dawley rats (180–220 g) were randomly assigned to two initial groups: a control group (N = 10) and a model group (N = 40). Nephropathy was induced in the model group via a single tail-vein injection of adriamycin (4 mg/kg) (12). On day 10 post-induction, 24-hour urinary protein was measured to confirm successful modeling. The model group was subsequently randomized into four subgroups: the Model group; a low-dose MHCD group (MHCD-L, 6.25 g/kg/day); a medium-dose MHCD group (MHCD-M, 12.5 g/kg/day); and a high-dose MHCD group (MHCD-H, 25 g/kg/day). The total amount of crude herbs in the clinical prescription was 120 g/day. Assuming a 60-kg adult, the human crude-herb dose was 2 g/kg/day. Using standard body-surface-area conversion, the rat-equivalent dose was approximately 12.5 g/kg/day, which was used as the medium dose; 6.25 g/kg/day and 25 g/kg/day were therefore set as the low and high doses, respectively, to evaluate dose dependence. All treatments were administered by oral gavage for six consecutive weeks. Rats in the control and model subgroups received an equivalent volume of purified water.

Twenty-four-hour urine samples were collected during the weekends of weeks 2, 4, and 6 post-modeling. Total urine volume was recorded, and 24-hour urinary protein excretion was quantified. Two hours after the final administration, rats were anesthetized with intraperitoneal injection of pentobarbital sodium (40 mg/kg), and blood was withdrawn from the abdominal aorta. Samples were allowed to clot at 37 °C for 2 hours and centrifuged at 3000 rpm for 10 minutes to obtain serum. Serum concentrations of urea nitrogen (Urea), creatinine (Scr), albumin (ALB), triglycerides (TG), and total cholesterol (TCH) were determined using a fully automated biochemical analyzer. Immediately afterward, kidneys were excised, and the renal cortex was isolated. One portion was fixed in 10% neutral buffered formalin for paraffin embedding; a second portion was fixed in 2.5% glutaraldehyde for electron microscopy; and the remaining tissue was flash-frozen in liquid nitrogen and stored at −80 °C for subsequent analyses. All procedures were approved by the Animal Ethics Committee of Xiyuan Hospital, China Academy of Chinese Medical Sciences (Approval No. 2024XLC036-3).

### 2.3 *In vitro* experiment on adriamycin-induced podocyte injury

For *in vitro* studies, conditionally immortalized mouse podocytes (MPC5) were cultured under permissive conditions (33 °C, humidified 5% CO_2_) in RPMI-1640 medium supplemented with 10% fetal bovine serum (FBS), 1% penicillin–streptomycin (dual antibiotics), and 10 U/mL recombinant mouse interferon-γ (IFN-γ). Cells were subcultured at a 1:3 ratio once they reached 70%–80% confluence. Differentiation was induced by switching to IFN-γ–free medium and transferring the cells to a 37 °C, 5% CO_2_ incubator for 10–14 days. All culture flasks and plates were pre-coated with rat tail collagen type I to promote adhesion and differentiation. Podocyte injury was induced by exposing differentiated cells to 0.1 μg/mL adriamycin for a specified duration.

### 2.4 Renal histopathology

#### 2.4.1 Histological staining

Renal tissue specimens were fixed in 10% neutral buffered formalin for at least 24 hours, dehydrated through graded ethanol, cleared in xylene, embedded in paraffin, and sectioned at 4 μm. Sections were stained with hematoxylin–eosin (HE), Masson’s trichrome, and periodic acid–Schiff (PAS) following standard protocols. After staining, sections were dehydrated through graded alcohol, cleared in xylene, and mounted using a synthetic resin medium. Glomerular pathological alterations across experimental groups were examined under a light microscope.

#### 2.4.2 Transmission electron microscopy

For ultrastructural analysis, renal cortical tissues were initially fixed in 2.5% glutaraldehyde for 2 hours at room temperature and subsequently stored in fresh fixative at 4 °C. Samples were washed twice with 0.1 M phosphate-buffered saline (PBS), post-fixed in 1% osmium tetroxide for 2 hours, and dehydrated through a graded ethanol series. Tissues were then infiltrated and embedded in Epon812 resin. After polymerization, semi-thin sections were cut and stained with toluidine blue for preliminary orientation. Ultra-thin sections were subsequently prepared using an ultramicrotome, mounted on copper grids, and stained with uranyl acetate (15 minutes) followed by lead citrate (10 minutes). The sections were examined under a transmission electron microscope to assess glomerular ultrastructure and podocyte foot process effacement.

#### 2.4.3 Immunohistochemistry

Paraffin-embedded renal tissue blocks were sectioned, baked, deparaffinized in xylene, and rehydrated through a graded ethanol series. After heat-induced antigen retrieval in citrate buffer, endogenous peroxidase activity was quenched and nonspecific binding was blocked with normal serum. Sections were incubated overnight at 4 °C with primary antibodies against FN, LN, and Col IV. Following thorough washing, an HRP-conjugated secondary antibody was applied and incubated at 37 °C for 60 minutes. Antigen–antibody complexes were visualized using 3,3′-diaminobenzidine (DAB), producing a brown precipitate. The sections were counterstained with hematoxylin, differentiated in acid alcohol, and blued in weak ammonia solution. Finally, they were dehydrated through graded ethanol, cleared in xylene, and mounted with neutral resin.

For quantitative evaluation, expression of FN, LN, and Col IV was measured by calculating the integrated optical density (IOD) of positive signals under a light microscope, defined as the product of average optical density and total positive area.

### 2.5 Western blot

Renal cortex tissues or cultured podocytes were homogenized and lysed in RIPA buffer containing 1% PMSF. After centrifugation, the supernatant was collected, and protein concentrations were determined using a BCA assay. Samples were diluted in 5× loading buffer, denatured at 100 °C for 10 minutes, and stored at −20 °C until use. SDS-PAGE gels were prepared according to target protein molecular weights. Protein markers and samples were sequentially loaded for electrophoresis, followed by membrane transfer and blocking. Membranes were incubated with primary antibodies overnight at 4 °C and washed four times with TBST (5 min each). Secondary antibodies were applied at room temperature for 2 hours, followed by four additional TBST washes. Bands were visualized using hypersensitive ECL reagents, and grayscale intensity was quantified using ImageJ.

### 2.6 Quantitative reverse transcription polymerase chain reaction

Total RNA from renal tissues and podocytes was extracted using TRIzol and reverse-transcribed into cDNA. Quantitative PCR was performed using a real-time PCR system, and relative mRNA expression was calculated using the 2^−ΔΔCt^ method. Primers were as follows: Arid5a: Forward 5′–ACAAGCCACTGCCTCCT–3′, Reverse 5′–CTGTTCTGTGCTGTCCCTC–3′; AIM2: Forward 5′–ACCTTTGGCACAGTAAC–3′, Reverse 5′–TAAACAGCCCATTCACA–3′; β–actin: Forward 5′–CATCCGTAAAGACCTCTATGCC–3′, Reverse 5′–ATGGAGCCACCGATCCACA–3′.

### 2.7 ASC oligomerization assay

For Triton fractionation, tissues or cells were lysed in Triton buffer and centrifuged at 6000 r/min for 15 minutes at 4 °C, yielding Triton X-100-soluble (supernatant) and -insoluble (pellet) fractions. The soluble fraction was mixed with 2× SDS loading buffer and heated at 95 °C for 5 minutes. The insoluble pellet was resuspended in Triton buffer and cross-linked with 2 mM DSS at 37 °C for 30 minutes, then centrifuged again at 6000 r/min for 15 minutes. The final pellet was dissolved in 2× SDS loading buffer. Samples were resolved by SDS-PAGE, transferred, and blocked. Membranes were incubated with an ASC antibody overnight at 4 °C, followed by secondary antibody incubation for 45 minutes at room temperature. Protein signals were detected using hypersensitive ECL and imaged with a gel documentation system.

### 2.8 ELISA

Podocytes were digested with trypsin and centrifuged at 500 r/min for 5 minutes at 4 °C to collect the supernatant. IL-1β (Catalog #ZC-37974) and IL-18 (Catalog #ZC-37973) levels were quantified using commercial ELISA kits (ZCIBIO Technology Co., Ltd.) following the manufacturer’s instructions, and absorbance was measured at 450 nm.

### 2.9 CCK-8 assay

Differentiated mature podocytes were seeded into 96-well plates and subjected to the designated experimental interventions. After treatment, the culture medium was removed and each well was supplemented with a 9:1 mixture of phenol red–free RPMI 1640 medium and CCK-8 solution. Plates were incubated at 37 °C with 5% CO_2_ for 1.5–2 hours, after which optical density (OD) was measured at 450 nm using a microplate reader.

### 2.10 Immunofluorescence

Podocyte coverslips were fixed in 4% paraformaldehyde for 15 minutes and permeabilized with 0.1% Triton X-100 for 30 minutes at room temperature. Following PBS washes, non-specific epitopes were blocked with 1% BSA. Cells were then incubated with the primary antibody overnight at 4 °C and subsequently with a fluorescently labeled secondary antibody for 60 minutes at room temperature. After application of anti-fade mounting medium, images were acquired using a fluorescence microscope.

### 2.11 Cell transfection

Differentiated mature podocytes were seeded into culture plates and transfected with Lipofectamine 3000 according to the manufacturer’s protocol. The expression constructs included an Arid5a-overexpression plasmid (pcDNA3.1, OE-Arid5a) and its corresponding empty vector control. Three independent siRNAs targeting Aim2 were used: Forward 5′-GGCAGAUAGGACAGAGUUATT-3′, Reverse 5′-UAACUCUGUCCUAUCUGCCTT-3′; si-AIM2-2: Forward 5′-GGAAGGAAGACAAGAGAUATT-3′, Reverse 5′-UAUCUCUUGUCUUCCUUCCTT-3′; si-AIM2-3: Forward 5′-GGAACAGGCUGCUACAGAATT-3′, Reverse 5′-UUCUGUAGCAGCCUGUUCCTT-3′ and negative control siRNA (siRNA-NC): Forward 5′-UUCUCCGAACGUGUCACGUTT-3′, Reverse 5′-ACGUGACACGUUCGGAGAATT-3′. Cells were harvested 48 hours post-transfection for subsequent analyses.

### 2.12 Electrophoretic mobility shift assay

The potential Arid5a binding site within the murine AIM2 promoter region was predicted using the JASPAR database. Based on this prediction, labeled wild-type (WT) probes, unlabeled WT competitor probes, and unlabeled mutant (MUT) probes were designed and synthesized. Nuclear proteins were extracted from podocytes, quantified, and used for EMSA. Binding reactions were assembled, electrophoresed, transferred to a membrane, and cross-linked. Following blocking and sequential incubations, signals were detected using an ECL method. Probe sequences were as follows: WT probe: Forward: 5′–CTGGTTCCTAATATTGATTCCCTCATC-3′; Reverse: 5′-GATGAGGGAATCAATATTAGGAACCAG-3′, MUT probe: Forward: 5′-CTGGTTCACGGCTACCAGCCTTTCATC-3′; Reverse: 5′-GATGAAAGGCTGGTAGCCGTGAACCAG-3′

### 2.13 Luciferase reporter assay

The murine AIM2 promoter was cloned into the pGL3-Basic vector to generate the AIM2(WT) luciferase reporter construct. A mutant promoter construct, AIM2(MUT), containing site-directed alterations at the predicted Arid5a binding motif, was generated as a control. A murine Arid5a expression plasmid was also constructed. Podocytes were co-transfected with the Arid5a expression plasmid and either AIM2(WT) or AIM2(MUT) reporter plasmids. At 48 hours post-transfection, luciferase activity was quantified using a dual-luciferase reporter assay according to the manufacturer’s instructions.

### 2.14 Statistical analysis

Statistical analyses were performed using SPSS version 23.0. Continuous variables were first assessed for normality. For normally distributed data, one-way analysis of variance (ANOVA) was used to compare multiple groups. When homogeneity of variance was met, *post-hoc* pairwise comparisons were conducted using the least significant difference (LSD) test; otherwise, Dunnett’s T3 test was applied. Non-parametric methods were used for data that did not follow a normal distribution. Normally distributed data are presented as mean ± standard deviation (SD), whereas non-normally distributed data are reported as median with interquartile range. A two-tailed P < 0.05 was considered statistically significant.

## 3 Results

### 3.1 *In vivo* experiments: MHCD modulates Arid5a, AIM2, and pyroptosis, and ameliorates renal injury in ADR rats

#### 3.1.1 MHCD attenuates proteinuria and preserves renal function in ADR rats

**Figure 1:**
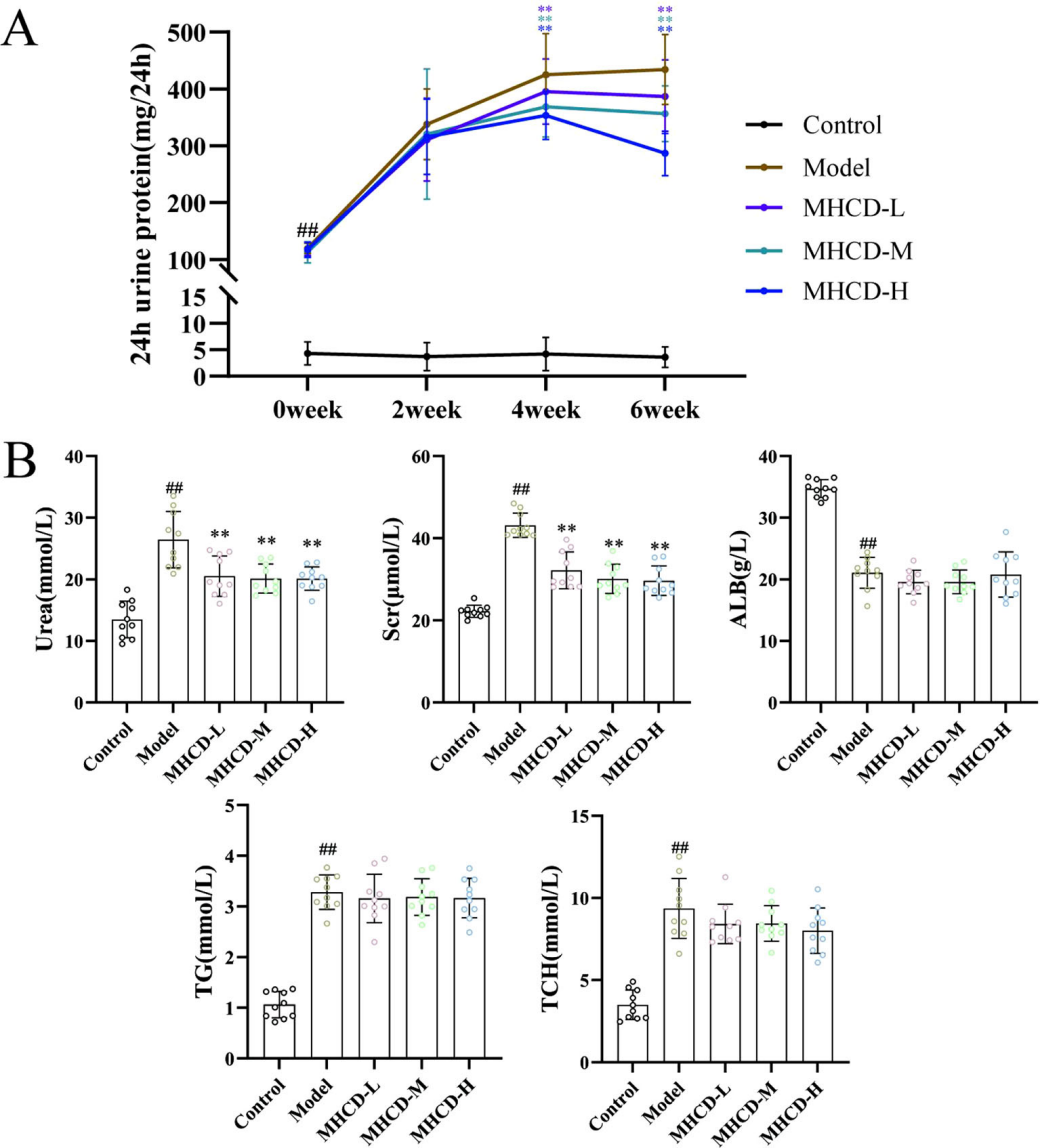
MHCD reduces proteinuria and improves blood biochemical indicators. **(A)** MHCD reduced proteinuria (n = 10); **(B)** Effect of MHCD on urea, Scr, ALB, TG, and TCH (n = 10). MHCD-L, low-dose MHCD; MHCD-M, medium -dose MHCD; MHCD-H, high -dose MHCD. ^##^*P* < 0.01 vs. control group; **P < 0.01 vs. model group.

At week 2, 24-h urinary protein levels in the MHCD groups remained comparable to those in the model group (**Figure 1A**). Between weeks 4 and 6, all MHCD dose groups exhibited a downward trend in 24-h urinary protein, with significant reductions compared with the model group (*P* < 0.01; **Figure 1A**). These findings indicate that MHCD reduces proteinuria across all doses, with the high-dose group showing the greatest effect.

After 6 weeks of treatment, serum biochemical parameters were assessed (**Figure 1B**). Relative to the control group, the model group displayed markedly elevated urea and serum creatinine (Scr) levels (*P* < 0.01). All MHCD dose groups showed reductions in both parameters compared with the model group. Serum albumin (ALB) was significantly decreased in the model group versus the control group (*P* < 0.01); however, MHCD did not significantly alter ALB levels at any tested dose (*P* > 0.05). Triglyceride (TG) and total cholesterol (TCH) levels were similarly elevated in the model group (*P* < 0.01), and MHCD administration did not significantly affect either parameter (*P* > 0.05). Overall, these results indicate that MHCD selectively improves serum biochemical abnormalities in ADR rats.

#### 3.1.2 MHCD ameliorates renal histopathological injury in ADR rats

**Figure 2:**
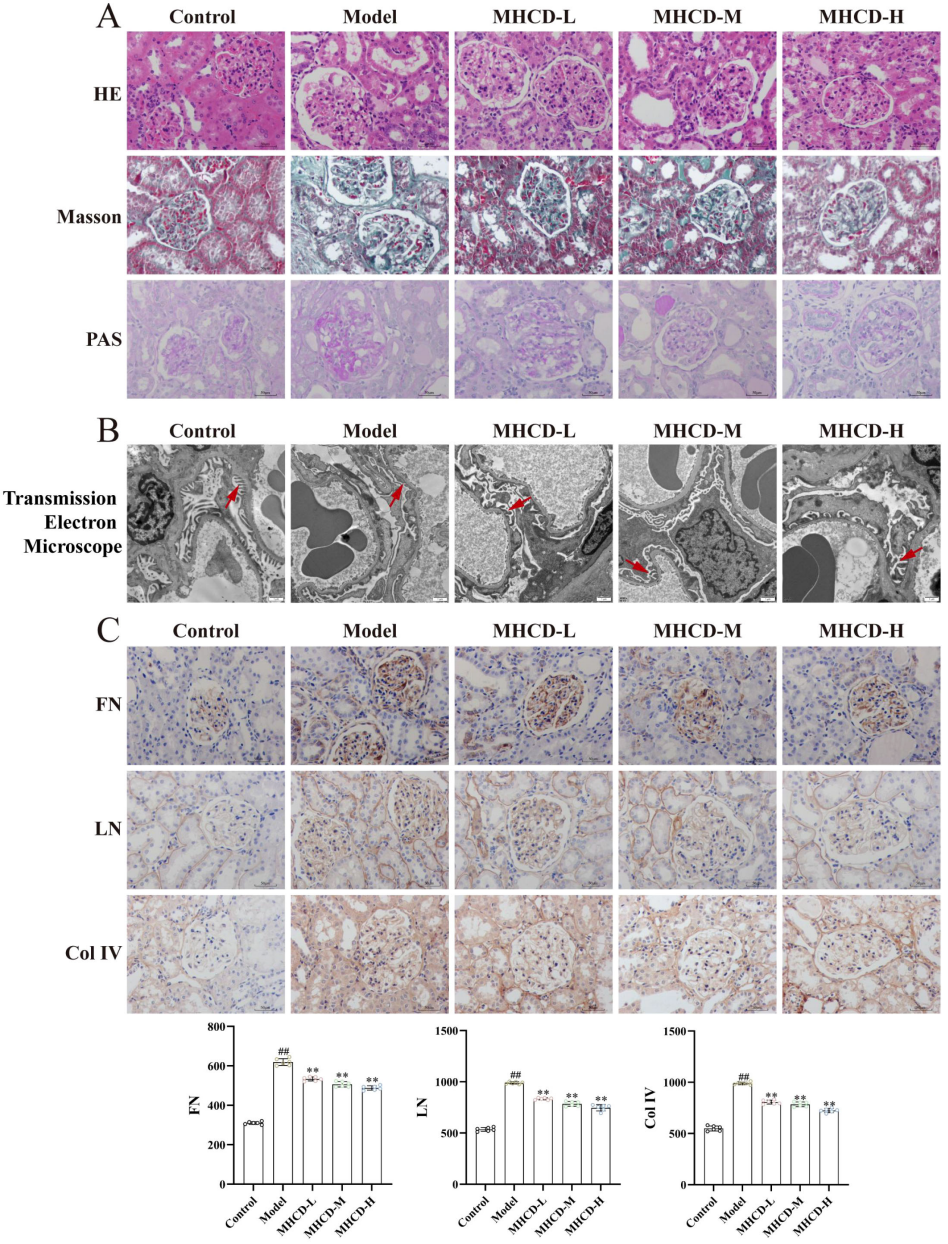
MHCD alleviates pathological renal injury in ADR rats. **(A)** HE, Masson’s trichrome staining and PAS staining to observe the effect of MHCD on renal pathological changes (200 ×); **(B)** Observation on the effect of MHCD on renal ultrastructure by transmission electron microscopy (8200 ×). Arrows indicate representative podocyte foot process effacement; **(C)** MHCD downregulated the expression of the renal fibrosis indices FN, LN, and Col IV (n = 6, 200 ×). MHCD-L, low-dose MHCD; MHCD-M, medium-dose MHCD; MHCD-H, high-dose MHCD. ^##^*P* < 0.01 vs. control group; **P < 0.01 vs. model group.

Kidney tissues from each group were evaluated using HE, Masson, and PAS staining (**Figure 2A**). In the control group, renal morphology appeared normal, with glomeruli displaying intact architecture, open capillary loops, and well-defined Bowman’s spaces, accompanied by orderly renal tubules lined with epithelial cells of normal morphology. In contrast, the model group exhibited pronounced pathological alterations, including compensatory glomerular hypertrophy or atrophy, mesangial cell and matrix proliferation, excessive collagen deposition, tubular disorganization with cystic dilation or atrophy, and marked epithelial swelling with hydropic degeneration. These histopathological abnormalities were ameliorated to varying degrees in the MHCD-treated groups. Ultrastructural changes assessed by transmission electron microscopy (**Figure 2B**) revealed extensive podocyte foot process effacement in the model group compared with the control group, although the glomerular basement membrane showed no obvious thickening. All MHCD dose groups demonstrated partial restoration of podocyte foot processes. Immunohistochemistry for fibrosis-related markers—fibronectin (FN), laminin (LN), and collagen IV (Col IV; **Figure 2C**)—showed elevated expression in the model group relative to controls, whereas MHCD administration reduced their levels. Collectively, these results indicate that MHCD mitigates renal histopathological injury in ADR-exposed rats.

#### 3.1.3 MHCD regulates Arid5a, AIM2, and pyroptosis in the renal tissues of ADR rats

**Figure 3:**
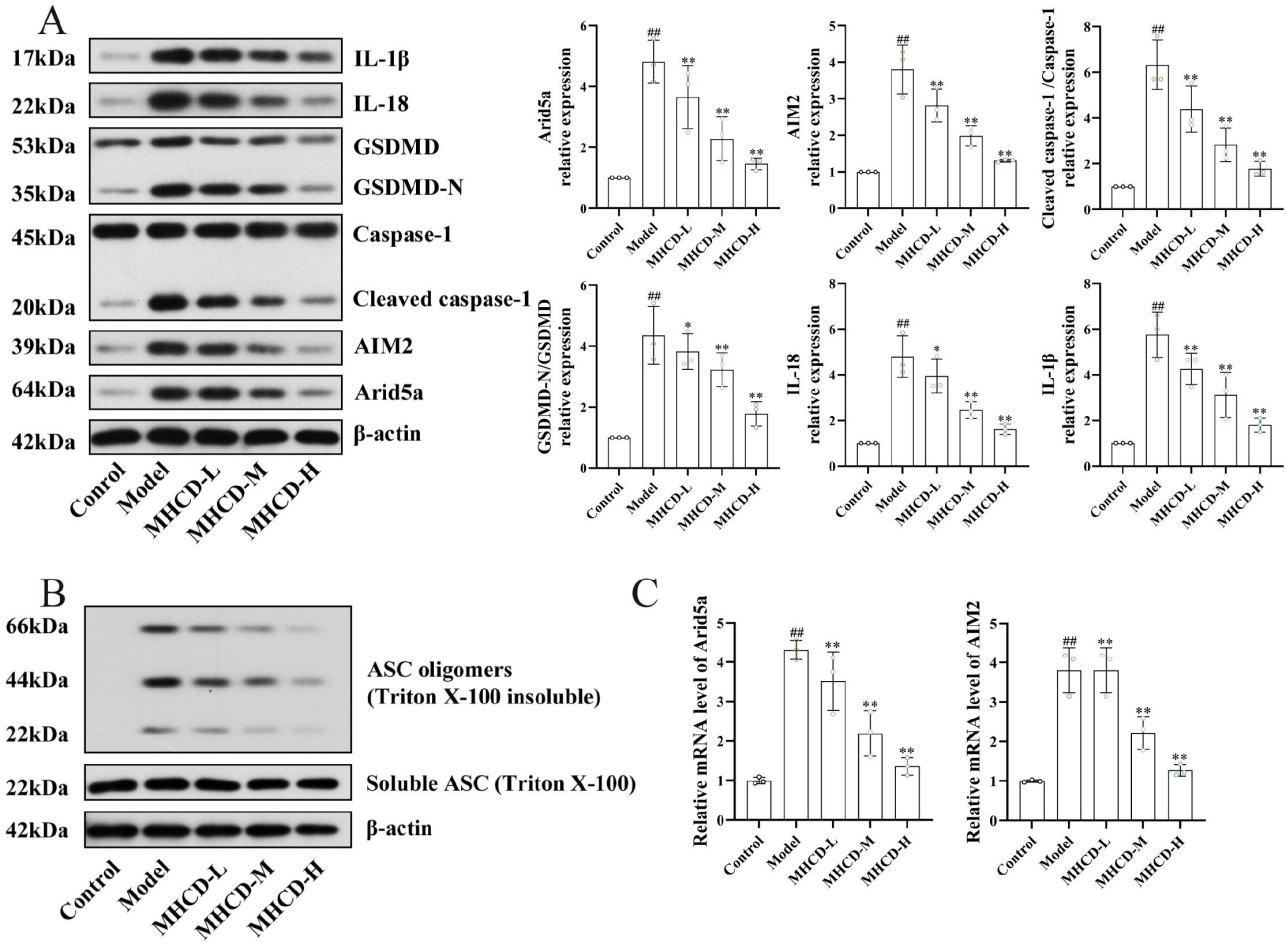
MHCD suppresses Arid5a and AIM2 expression and limits ASC oligomerization and pyroptosis in the renal tissues of ADR rats (n = 3). **(A)** Western blot analysis showing that MHCD inhibited the expression of Arid5a, AIM2, and pyroptosis-related proteins Caspase-1, GSDMD, IL-1β, and IL-18. **(B)** MHCD limited ASC oligomerization in the renal tissues of ADR rats. **(C)** RT-qPCR assay showed that MHCD inhibited the mRNA expression of Arid5a and AIM2. MHCD-L, low-dose MHCD; MHCD-M, medium-dose MHCD; MHCD-H, high-dose MHCD. ^##^*P* < 0.01 vs. control group; *P < 0.05 and **P < 0.01 vs. model group.

As shown in **Figure 3A**, the protein levels of IL-1β, IL-18, cleaved caspase-1, and GSDMD-N were markedly elevated in the model group relative to the control group (P < 0.01), confirming successful induction of adriamycin-triggered pyroptosis. MHCD treatment substantially reduced the expression of these pyroptosis-related proteins across all dose groups, exhibiting a dose-dependent trend (*P* < 0.05 or *P* < 0.01; **Figure 3A**). ASC oligomerization was also significantly increased in the model group (**Figure 3B**), and both Western blot and RT-qPCR demonstrated upregulated AIM2 expression (**Figures 3A, C**). MHCD administration suppressed ASC oligomerization and AIM2 expression, indicating inhibition of inflammasome assembly and activation. Additionally, Western blot and RT-qPCR showed marked upregulation of Arid5a in the model group (*P* < 0.01), which decreased in a dose-dependent manner following MHCD treatment (**Figures 3A, C**). Together, these findings suggest that MHCD suppresses Arid5a and AIM2 expression, thereby reducing caspase-1 activation, GSDMD cleavage, and IL-1β/IL-18 release—changes consistent with attenuated podocyte pyroptosis and reduced renal inflammatory injury.

### 3.2 *In vitro* experiments: Arid5a enhances podocyte pyroptosis and increases AIM2 expression through transcriptional regulation

#### 3.2.1 Construction of podocyte Arid5a overexpression and AIM2 knockdown

**Figure 4:**
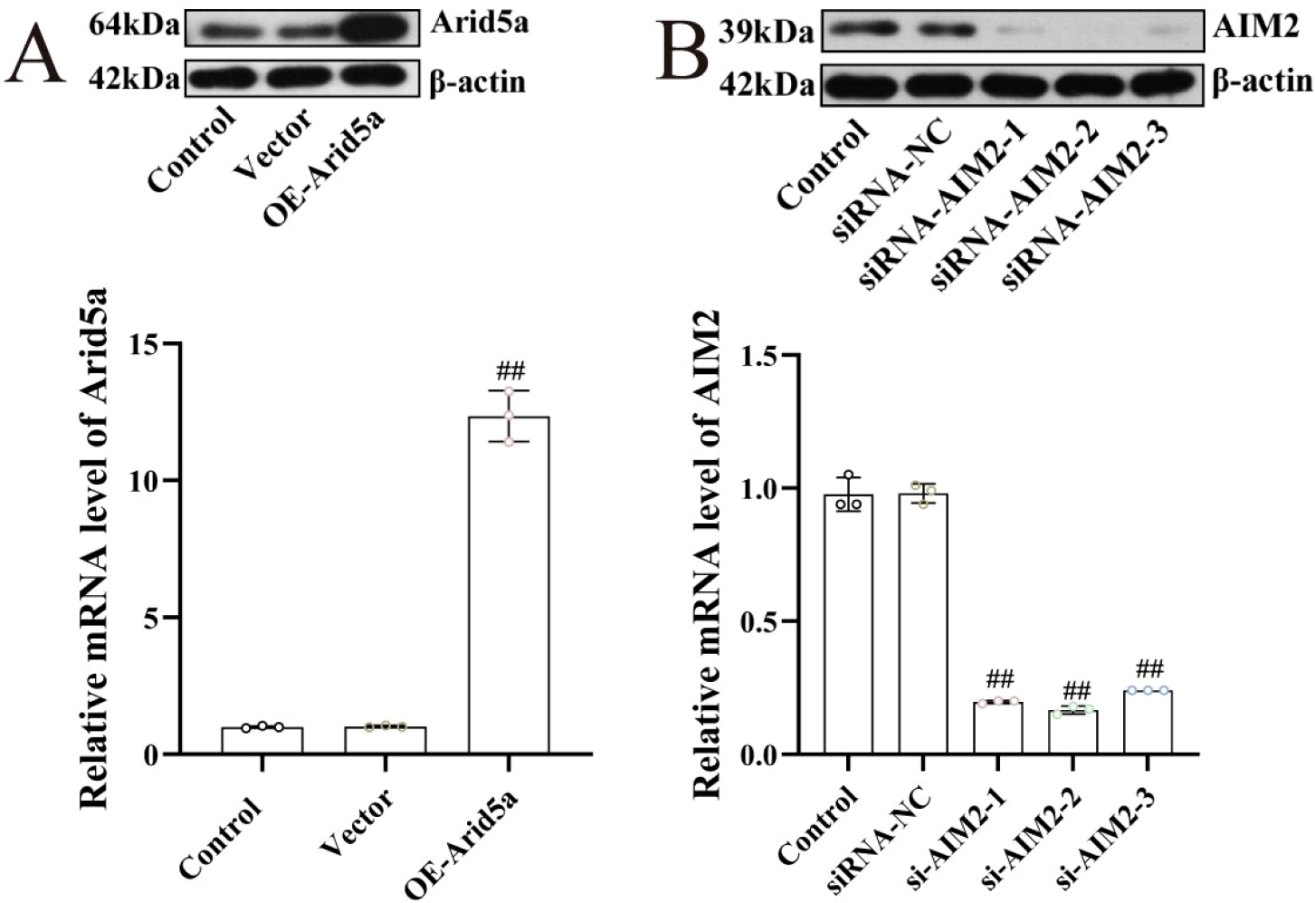
Construction of podocyte with Arid5a overexpression and AIM2 knockdown (n = 3). **(A)** Verification of Arid5a transfection efficiency by western blot and RT-qPCR assays. **(B)** Verification of si-AIM2 transfection efficiency by western blot and RT-qPCR assays. ^##^*P* < 0.01 vs vector group and siRNA-NC group.

Overexpression plasmids for Arid5a and corresponding negative control plasmids were constructed and transfected into podocytes, with transfection efficiency verified by Western blot and RT-qPCR (**Figure 4A**). For AIM2 knockdown, three siRNAs (si-AIM2-1, si-AIM2-2, and si-AIM2-3) and a negative control siRNA (siRNA-NC) were designed and synthesized. Western blot and RT-qPCR were used to assess knockdown efficiency, and si-AIM2–2 was selected for subsequent experiments (**Figure 4B**).

#### 3.2.2 Arid5a overexpression increases the expression of AIM2 and pyroptosis-related molecules

**Figure 5:**
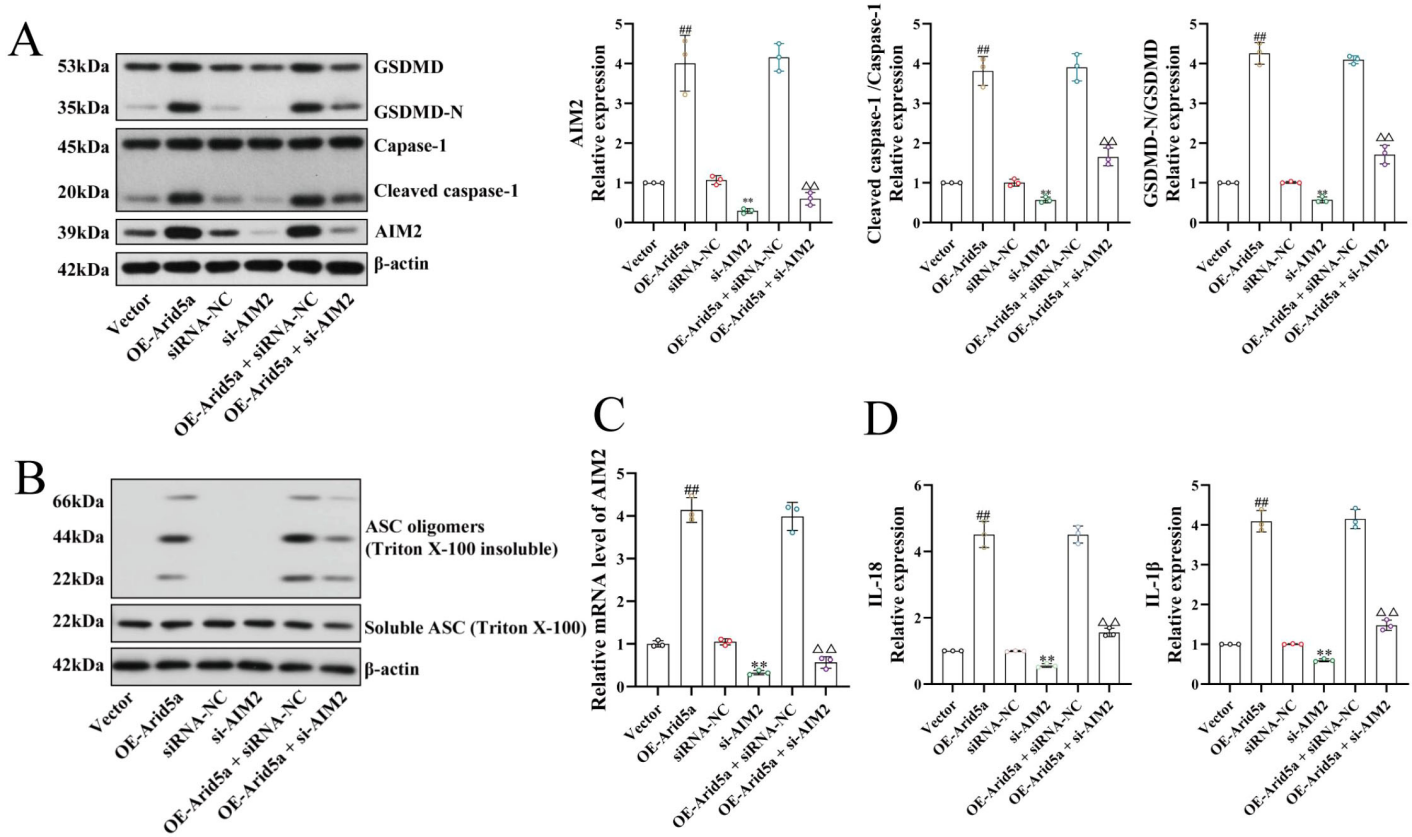
Arid5a promotes podocyte pyroptosis by upregulating AIM2 (n = 3). **(A)** Western blot showing that Arid5a overexpression upregulated the expression of AIM2, Caspase-1, and GSDMD. **(B)** Arid5a overexpression upregulated ASC oligomerization. **(C)** RT-qPCR assay showing that Arid5a overexpression upregulated the mRNA expression of AIM2. **(D)** ELISA showing that Arid5a overexpression upregulated the levels of IL-1β and IL-18 in the cell supernatant. ^##^*P* < 0.01 vs. vector group; ^**^P < 0.01 vs. siRNA-NC group; ^△△^*P* < 0.01 vs. OE-Arid5a + siRNA-NC group. No significant difference was observed between the OE-Arid5a and OE-Arid5a + siRNA-NC groups.

In podocytes overexpressing Arid5a, both AIM2 mRNA and protein levels were significantly elevated (P < 0.01; **Figures 5A, C**). Western blot analysis further revealed increased levels of cleaved caspase-1 and GSDMD-N (P < 0.01; **Figure 5A**), accompanied by enhanced ASC oligomerization (**Figure 5B**). ELISA results showed significant increases in IL-1β and IL-18 secretion into the culture supernatant (P < 0.01; **Figure 5D**). These data demonstrate that Arid5a overexpression upregulates AIM2 expression, enhances caspase-1 activation, promotes GSDMD cleavage, and increases IL-1β/IL-18 release—changes indicative of intensified pyroptotic activity in podocytes.

#### 3.2.3 AIM2 knockdown partially reduces pyroptosis in Arid5a-overexpressing podocytes

To determine whether Arid5a-induced enhancement of pyroptosis is mediated through the AIM2 inflammasome, AIM2 was silenced using siRNA. AIM2 knockdown markedly reduced cleaved caspase-1, GSDMD-N, and ASC oligomerization (**Figures 5A, B**), and IL-1β/IL-18 release (P < 0.01; **Figure 5D**). When AIM2 knockdown was combined with Arid5a overexpression (Arid5a + si-AIM2 group), these pyroptosis-related alterations were substantially reversed. Taken together, these findings indicate that silencing AIM2 significantly attenuates pyroptotic activity in Arid5a-overexpressing podocytes, supporting a functional link between Arid5a-mediated effects and AIM2 inflammasome activation.

#### 3.2.4 Arid5a regulates AIM2 expression through its transcriptional activity

**Figure 6:**
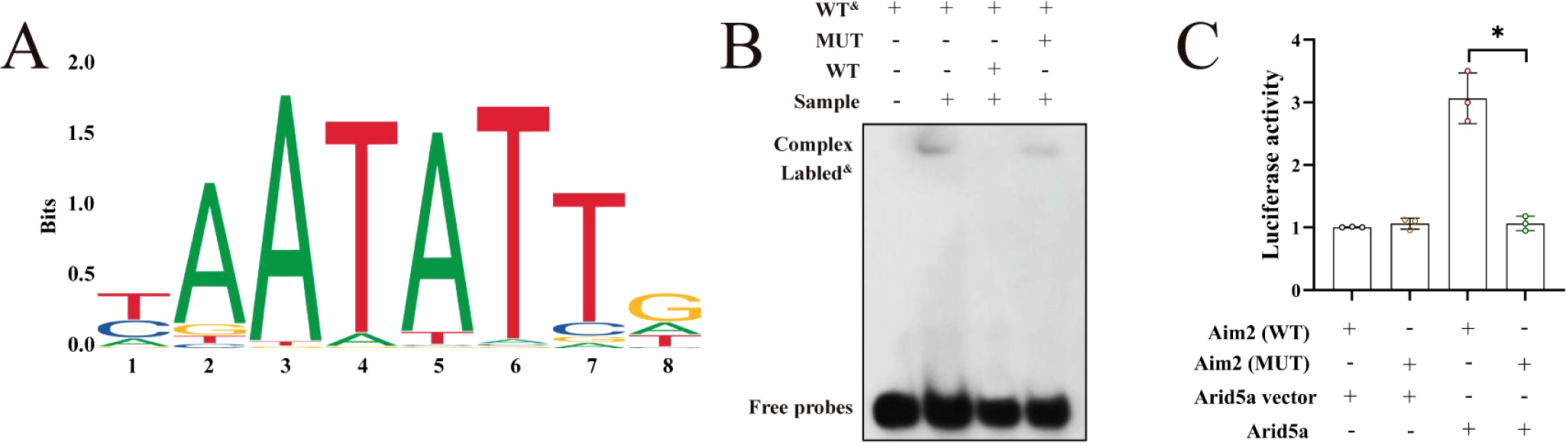
Arid5a regulates AIM2 expression through its transcription factor activity. **(A)** The binding sites between Arid5a and the AIM2 promoter region were predicted using the JASPAR database. **(B)** EMSA for Arid5a binding to the AIM2 promoter. **(C)** Luciferase reporter assay for Arid5a binding to the AIM2 promoter (n = 3). WT^&^, Labeled wild-type probe; WT, wild-type; MUT, mutant. ^*^P < 0.05 vs. mutant group.

Binding sites for Arid5a within the AIM2 promoter were predicted using the JASPAR database (**Figure 6A**). Wild-type (WT) and mutant (MUT) AIM2 promoter constructs harboring the predicted binding motif were generated for EMSA and luciferase reporter assays. In the EMSA assay, addition of an unlabeled AIM2 WT probe markedly diminished the intensity of the shifted band, whereas the unlabeled AIM2 MUT probe produced no appreciable effect (**Figure 6B**). Consistently, luciferase assays demonstrated that Arid5a significantly enhanced reporter activity driven by the AIM2 WT promoter, while mutation of the predicted binding site abolished this Arid5a-dependent activation (**Figure 6C**). These findings collectively demonstrate that Arid5a directly binds the AIM2 promoter to activate its transcription, thereby increasing AIM2 expression.

### 3.3 *In vitro* experiments: MHCD mitigates podocyte pyroptosis by suppressing the Arid5a/AIM2 signaling axis

**Figure 7:**
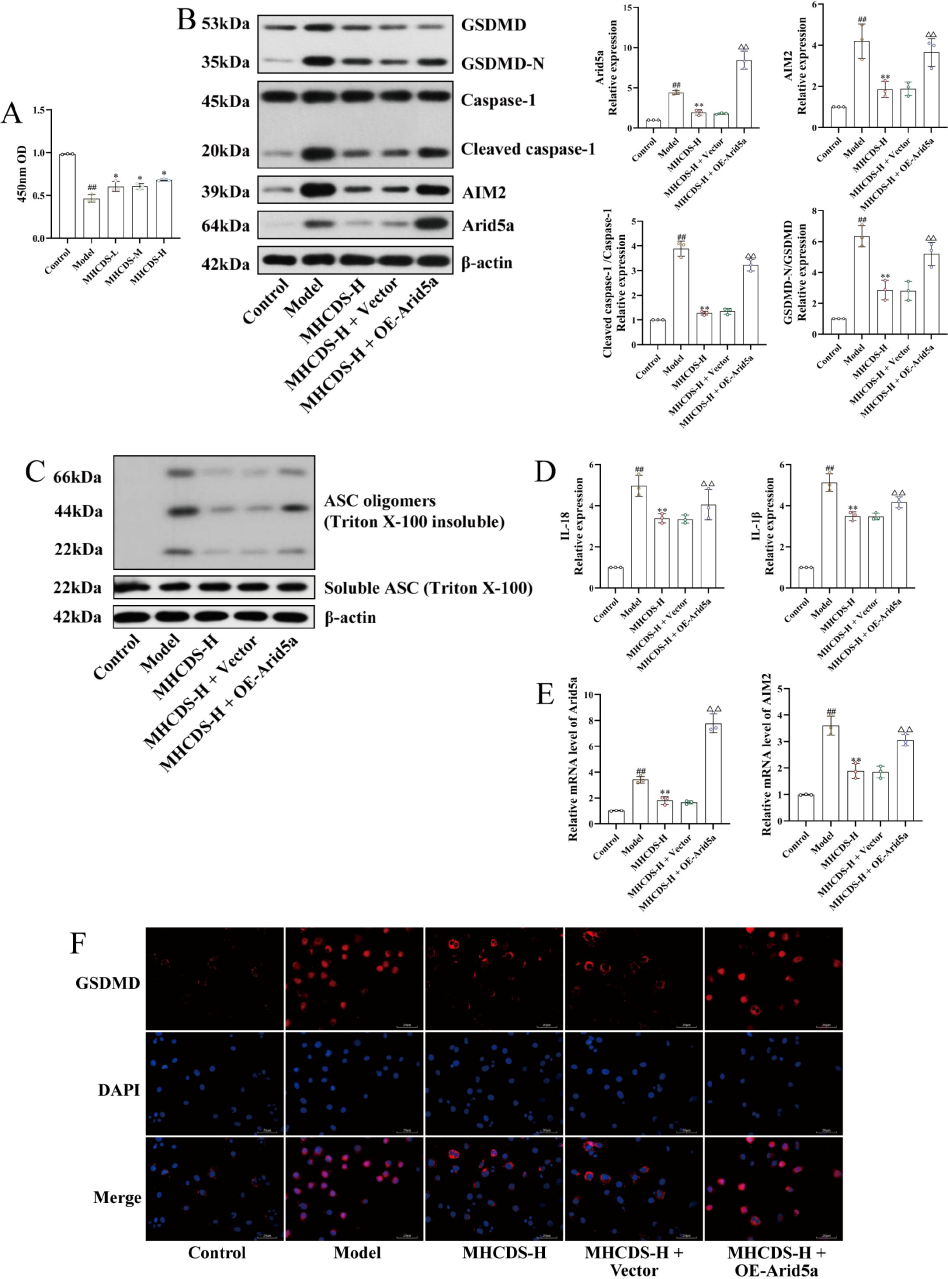
MHCD attenuates podocyte pyroptosis by inhibiting the Arid5a/AIM2 signaling axis (n = 3). **(A)** CCK-8 assay showing that MHCDS protected podocyte viability in a dose-dependent manner. **(B)** Western blot showing that MHCDS inhibited the expression of Arid5a, AIM2, and pyroptosis-related proteins Caspase-1 and GSDMD. **(C)** MHCDS inhibited ASC oligomerization in podocytes. **(D)** ELISA showing that MHCDS inhibited the levels of IL-1β and IL-18 in the cell supernatant. **(E)** RT-qPCR assay showing that MHCDS inhibited the mRNA expression of Arid5a and AIM2. **(F)** Immunofluorescence staining of GSDMD. MHCDS-L, low-dose MHCD-containing serum, MHCDS-M, medium-dose MHCD-containing serum, MHCDS-H, high-dose MHCD-containing serum. ^##^*P* < 0.01 vs. control group; **P < 0.01 vs. Model group; ^△△^*P* < 0.01 vs. MHCDS-H+ vector group.

Podocyte injury was induced with adriamycin (0.1 μg/mL), after which cells were treated with MHCD-containing serum. Cell viability, measured using the CCK-8 assay, was significantly reduced in the model group compared with controls (*P* < 0.01; **Figure 7A**). Treatment with low-, medium-, and high-dose MHCD-containing serum increased podocyte viability relative to the model group (*P* < 0.05). Viability did not differ significantly between medium- and high-dose groups (*P* > 0.05), although the high-dose group showed a significant improvement over the low-dose group (*P* < 0.05). Because high-dose MHCD produced the most pronounced protective effect, it was used in subsequent mechanistic studies.

To define the role and mechanism of MHCD in regulating podocyte pyroptosis, we examined the expression of the Arid5a/AIM2 signaling axis and its downstream effectors. Relative to the control group, the model group exhibited marked increases in both mRNA and protein levels of Arid5a and AIM2 (*P* < 0.01; **Figures 7B, E**). Correspondingly, levels of cleaved caspase-1, GSDMD-N, and ASC oligomerization were significantly elevated (*P* < 0.01; **Figures 7B, C**), indicating activation of adriamycin-induced pyroptosis. ELISA assays further revealed substantial increases in IL-1β and IL-18 secretion (*P* < 0.01; **Figure 7D**). High-dose MHCD-containing serum markedly suppressed Arid5a and AIM2 expression at both the mRNA and protein levels and significantly reduced cleaved caspase-1, GSDMD-N, and ASC oligomerization (*P* < 0.01). Consistent decreases in IL-1β and IL-18 release were observed (*P* < 0.01), and immunofluorescence staining confirmed reduced GSDMD expression (**Figure 7F**). Mechanistic reversal experiments demonstrated that Arid5a overexpression partially attenuated the inhibitory effects of high-dose MHCD-containing serum, resulting in increased AIM2 expression, caspase-1 activation, GSDMD cleavage, ASC oligomerization, and IL-1β/IL-18 production relative to the MHCD-only group (*P* < 0.01; **Figures 7B–F**).

Collectively, these results show that MHCD inhibits podocyte pyroptosis at least in part by downregulating the transcription factor Arid5a, thereby suppressing AIM2 pathway activation, reducing caspase-1 activation and GSDMD cleavage, and ultimately limiting IL-1β and IL-18 release. The renoprotective effects of MHCD appear to depend, at least partially, on modulation of the Arid5a/AIM2 signaling axis.

## 4 Discussion

ADR rats constitute a widely used experimental model of CKD. In the early phase, this model primarily manifests podocyte injury resembling minimal change disease (MCD), whereas in later stages it progresses to pathological features characteristic of focal segmental glomerulosclerosis (FSGS). The temporal progression of renal injury in ADR rats broadly parallels the pathological evolution observed in chronic glomerulonephritis (19, 20). Across proteinuric kidney diseases—including MCD and FSGS—effacement or loss of podocyte foot processes is a defining histopathological feature. Injury, mutation, or depletion of podocytes critically disrupts the glomerular filtration barrier, permitting substantial leakage of plasma proteins into the urinary space and resulting in proteinuria. Notably, the severity of proteinuria correlates closely with the extent of foot-process effacement (21, 22). Accordingly, the present study employed both an adriamycin-induced nephropathy model and an *in vitro* podocyte injury model to systematically assess the protective effects of MHCD on podocyte damage. MHCD markedly attenuated proteinuria and improved renal functional parameters in ADR rats. Consistent with these functional benefits, histological and ultrastructural analyses demonstrated that MHCD reduced renal pathological alterations and ameliorated podocyte injury. Because renal fibrosis—defined by excessive extracellular matrix accumulation—represents the final common pathway of nearly all forms of CKD, its modulation is therapeutically meaningful. Although transient matrix deposition may assist early tissue repair, persistent accumulation progressively undermines renal architecture, culminating in irreversible functional decline (23). In this study, MHCD decreased renal expression of the fibrosis-related markers fibronectin (FN), laminin (LN), and collagen IV (Col IV), indicating a significant attenuation of fibrotic progression.

Renal inflammation is a highly complex, multifactorial process initiated by local or systemic insults such as uremic toxins, oxidative stress, and endotoxins. While initially adaptive, sustained inflammatory activation exacerbates renal injury and promotes the onset of renal fibrosis (24). Pyroptosis—a form of programmed cell death driven by inflammasome activation—has emerged as a critical contributor to renal injury and a rapidly expanding focus of kidney research (25). Canonical pyroptosis involves inflammasome assembly, caspase-1 activation, cleavage of gasdermin D (GSDMD), and release of pro-inflammatory cytokines IL-1β and IL-18. Upon exposure to diverse danger signals, inflammasome sensors recruit and activate caspase-1, which subsequently processes pro–IL-1β and pro–IL-18 and cleaves GSDMD to generate its pore-forming N-terminal fragment, ultimately inducing cell lysis and pyroptotic death. Podocytes, indispensable for maintaining glomerular filtration barrier integrity, are particularly vulnerable to injury. Their loss—through apoptosis, dysregulated autophagy, or pyroptosis—directly compromises barrier function and leads to proteinuria (26, 27). The AIM2 inflammasome, a cytosolic sensor of double-stranded DNA, activates caspase-1 and mediates IL-1β and IL-18 maturation as well as GSDMD cleavage, thereby initiating pyroptosis (28). Recent evidence indicates that AIM2 also plays a pivotal role in non-infectious renal injury (29). For example, Zhou et al. demonstrated marked AIM2 activation in aldosterone-induced renal injury (30), while Chi et al. reported elevated AIM2 expression in glomerular cells—including podocytes—where it drives inflammation and cellular damage via the caspase-1/IL-18 pathway (31). The present findings align with these reports and further underscore the importance of AIM2-mediated pyroptosis in podocyte structural and functional impairment. MHCD treatment not only reduced proteinuria and improved renal histopathology but also lowered expression levels of AIM2 and downstream pyroptotic mediators including caspase-1, ASC oligomers, GSDMD, IL-1β, and IL-18. Collectively, these results suggest that the renoprotective and podocyte-protective effects of MHCD may be mediated, at least in part, through suppression of the AIM2-dependent pyroptotic signaling pathway.

Arid5a was initially characterized as a DNA/RNA-binding protein with both transcriptional and post-transcriptional regulatory functions. Emerging evidence shows that, under inflammatory conditions, Arid5a translocates from the nucleus to the cytoplasm, where it binds mRNAs encoding key pro-inflammatory cytokines—including IL-6, Stat3, and T-bet—and enhances their stability, thereby amplifying inflammatory responses (32). In renal research, Li et al. demonstrated in an autoantibody-mediated glomerulonephritis model that Arid5a expression is markedly upregulated in both human and murine kidneys. Notably, Arid5a-deficient mice exhibited attenuated renal injury and inflammatory infiltration, accompanied by reduced expression of IL-17–related genes (17). Similarly, Tanaka et al. showed in an LPS-induced kidney injury model that the Arid5a/IL-6/PAI-1 signaling axis drives inflammatory and fibrotic progression, and that Arid5a loss partially suppresses IL-6 and PAI-1 upregulation (18). In this study, we identified Arid5a as a regulator of AIM2 expression; however, the protein-level mechanisms underlying their interaction required clarification. Bioinformatic analysis combined with JASPAR prediction revealed putative Arid5a-binding sites within the AIM2 promoter. Accordingly, we constructed WT and mutant AIM2 (MUT) promoter reporter plasmids and performed EMSA and luciferase assays. These experiments confirmed that Arid5a directly binds the AIM2 promoter and enhances its transcriptional activity, thereby upregulating AIM2 expression. Collectively, our findings suggest that Arid5a not only exerts pro-inflammatory functions but also serves as a molecular bridge linking upstream inflammatory cues to downstream inflammasome activation in renal injury. Furthermore, rescue experiments demonstrated that MHCD mitigates renal and podocyte injury at least in part by modulating the Arid5a/AIM2 pyroptotic signaling axis.

Previous studies from our group demonstrated that MHCD attenuates renal inflammatory injury by suppressing upstream inflammatory pathways, including TLR4/NF-κB-related signaling (14). In the present study, we specifically investigated the direct transcriptional regulation of AIM2 by Arid5a and demonstrated that Arid5a binds to the AIM2 promoter and enhances its transcriptional activity. These findings suggest that the inhibitory effect of MHCD on the Arid5a/AIM2 axis may also involve upstream anti-inflammatory signaling events, including NF-κB-related regulation. In addition, other dsDNA-sensing pathways, such as cGAS-STING, may also participate in renal inflammatory responses. However, these mechanisms were beyond the scope of the current study and were therefore not directly examined. Further studies are needed to determine whether MHCD exerts broader regulatory effects on upstream inflammatory signaling or DNA-sensing immune pathways in podocytes.

Compared with findings from previous studies, our work offers several novel insights. First, to our knowledge, we provide the first evidence in a podocyte pyroptosis model that Arid5a directly regulates AIM2 transcription. Second, we identify MHCD as the first traditional Chinese herbal formula capable of modulating pyroptosis through targeted regulation of the Arid5a/AIM2 signaling axis. These findings deepen the mechanistic understanding of MHCD’s anti-proteinuric and podocyte-protective effects and establish a theoretical foundation for developing new herbal or small-molecule therapeutics targeting the Arid5a/AIM2 pyroptotic pathway.

Despite these advances, several limitations warrant discussion. (i) Arid5a knockout animal models were not employed, preventing definitive establishment of its causal role in mediating MHCD’s effects. (ii) As a multi-component formula, the specific bioactive constituents of MHCD that regulate the Arid5a/AIM2 axis remain to be identified. (iii) Crosstalk between pyroptosis and other cell death pathways—including apoptosis and autophagy—likely exists and requires further elucidation. (iv) Clinical validation is still needed to determine whether Arid5a and AIM2 expression correlates with proteinuria severity or renal functional decline in patients. Future work combining RNA-seq, ChIP-seq, and other high-throughput approaches may help construct a comprehensive regulatory network of MHCD and identify critical molecular targets. Integrating mechanistic studies with clinical sample validation will facilitate the translation of MHCD from experimental investigation toward precision clinical application.

**Figure 8:**
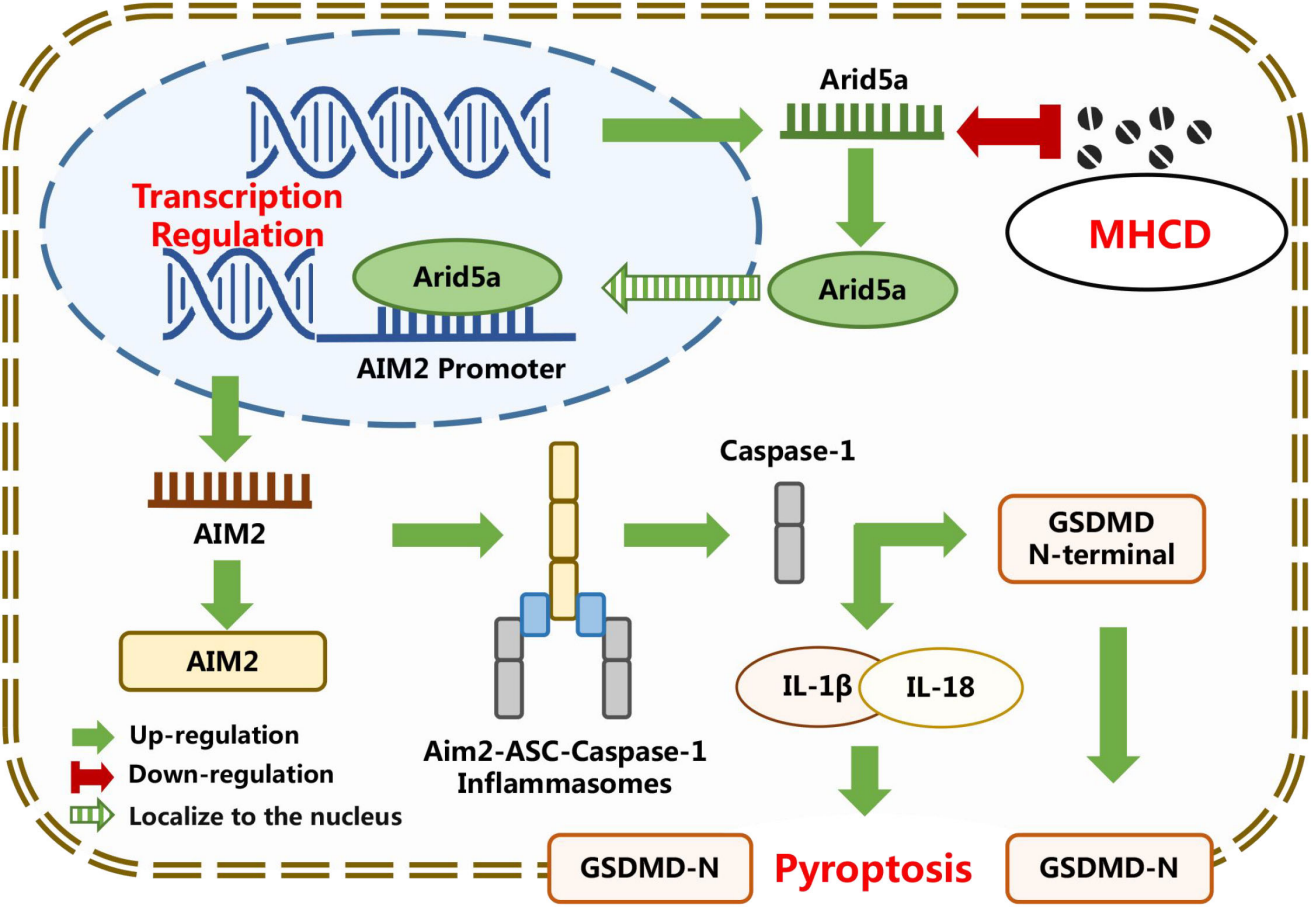
Schematic illustration of the proposed mechanism. Adriamycin-induced nephropathy and podocyte injury models were established to simulate the progression of CKD-associated proteinuria. Based on the identified regulatory effects of MHCD on Arid5a, AIM2, and pyroptosis, mutagenesis of the Arid5a-binding sites within the AIM2 promoter was performed, followed by EMSA and luciferase reporter assays to validate the mechanism by which Arid5a binds to the AIM2 promoter and regulates its transcription. Rescue experiments further demonstrated that MHCD inhibits podocyte pyroptosis through the modulation of the Arid5a/AIM2 signaling axis, providing new experimental evidence supporting the potential clinical application of MHCD in the treatment of CKD-related proteinuria.

In summary, building on the observed renoprotective and podocyte-protective effects of MHCD, our findings demonstrate that MHCD reduces Arid5a expression, interferes with its transcriptional regulatory activity, and suppresses AIM2 inflammasome assembly and activation. These effects inhibit caspase-1–mediated GSDMD cleavage and limit the release of pro-inflammatory cytokines IL-1β and IL-18 (**Figure 8**). Collectively, the results indicate that MHCD mitigates renal and podocyte injury, at least in part, through modulation of Arid5a-dependent transcriptional regulation of the AIM2 signaling axis and subsequent restraint of inflammasome-driven pyroptosis.

## Data Availability

The raw data supporting the conclusions of this article will be made available by the authors, without undue reservation.
